# Bringing genetics to heretofore intractable obligate intracellular bacterial pathogens: *Chlamydia* and beyond

**DOI:** 10.1371/journal.ppat.1010669

**Published:** 2022-07-28

**Authors:** Magnus Ölander, Barbara S. Sixt

**Affiliations:** 1 Department of Molecular Biology, Umeå University, Umeå, Sweden; 2 The Laboratory for Molecular Infection Medicine Sweden (MIMS), Umeå University, Umeå, Sweden; 3 Umeå Centre for Microbial Research (UCMR), Umeå University, Umeå, Sweden; Duke University School of Medicine, UNITED STATES

## What are obligate intracellular bacteria and why should we care?

As their designation implies, obligate intracellular bacteria are microbes that have developed lifestyles so closely entwined with the cells of the hosts they infect that they can reproduce only within the confines of these cells. Among this group of bacteria are several pathogenic species with a significant impact on human health. For instance, *Chlamydia* spp. are responsible for millions of cases of urogenital, ocular, and respiratory infections every year [1]. Moreover, *Coxiella burnetii* is the agent of Q fever [2], whereas members of the order *Rickettsiales* (specifically the genera *Rickettsia*, *Ehrlichia*, and *Anaplasma*) cause life-threatening vector-borne diseases, such as spotted fever and typhus [3].

While these pathogens are all restricted to life in an intracellular niche, they differ in the ways they reproduce and engage with their host cells. *Chlamydia* spp., *Ehrlichia* spp., *Anaplasma* spp., and *C*. *burnetii* have biphasic life cycles, in which the bacteria alternate between infectious (environmentally stable) and replicative (metabolically highly active) forms inside specialized membrane-enclosed vacuoles [1–3]. In contrast, *Rickettsia* spp. escape their vacuoles after uptake and replicate in the host cytosol without undergoing developmental transitions [3]. The various obligate intracellular lifestyles are enabled by distinct sets of virulence factors, which, for instance, engage in the subversion of host defenses and the hijacking of host resources and machineries.

Studying the molecular basis of these intimate relationships between host and bacteria can uncover unknown aspects of host–pathogen interactions and new targets for pharmacological intervention to help relieve the significant disease burden of intracellular infections. However, historically, the study of obligate intracellular bacteria was neglected due to the impeding lack of tools enabling their molecular genetic manipulation. While whole-genome sequencing provided the opportunity to predict their virulence traits, in the absence of genetic tools, direct links to specific bacterial genes could not be formally demonstrated.

Fortunately, recent years have seen a significant expansion of our genetic toolbox for these important pathogens. Here, we summarize these developments, with a focus on *Chlamydia* spp., along with a brief overview for other obligate intracellular bacteria. Furthermore, we provide examples of insights into chlamydial biology enabled by the expanded toolbox, and we conclude with a discussion on future perspectives for the molecular genetic manipulation of this group of bacteria.

## Why is the genetic manipulation of obligate intracellular bacteria so challenging?

A major factor restricting the genetic tractability of obligate intracellular bacteria is their main defining characteristic, i.e., their intracellular lifestyle. Delivery of DNA into bacteria residing within host cells is difficult. Therefore, most approaches that depend on DNA delivery require the bacteria first to be isolated before they can be modified extracellularly and then reintroduced into suitable host cells [4–7]. However, the distinct developmental forms of some species can be differentially receptive for DNA uptake, with the infective forms being generally considered less amenable to modification due to their rigid cell walls, condensed nucleoids, and/or reduced metabolic activities [8,9].

As a consequence of the resulting low transformation efficiencies, selection and recovery of modified bacteria typically requires the expansion of a very low number of transformed bacteria through passage in cell culture, a process that can take days to weeks. Notably, antibiotic resistance genes are commonly used as selection markers, but the list of antibiotics that can be used is limited by regulatory prohibitions. Moreover, antibiotics that cannot access the intracellular site of bacterial replication in an active form, or induce spontaneous resistance at a high frequency, cannot be used. On an encouraging note, several nonantibiotic selection markers, for instance, based on herbicide resistance or complementation of amino acid auxotrophies, have already been introduced in some obligate intracellular species [10–12].

The strict intracellular lifestyle also complicates the recovery of clonal strains of modified bacteria because in the absence of a possibility for host cell-free cultivation, clones cannot be obtained simply by picking colonies from agar plates. Plaque purification is applicable to certain obligate intracellular bacteria, such as some *Chlamydia* and *Rickettsia* spp. [13,14]. However, when plaque purification is not possible, clonal strains instead need to be recovered by the slow process of limiting dilution [15] or by technically more demanding techniques, such as micromanipulation [16], laser microdissection [17], or cell sorting [18].

**Fig 1:**
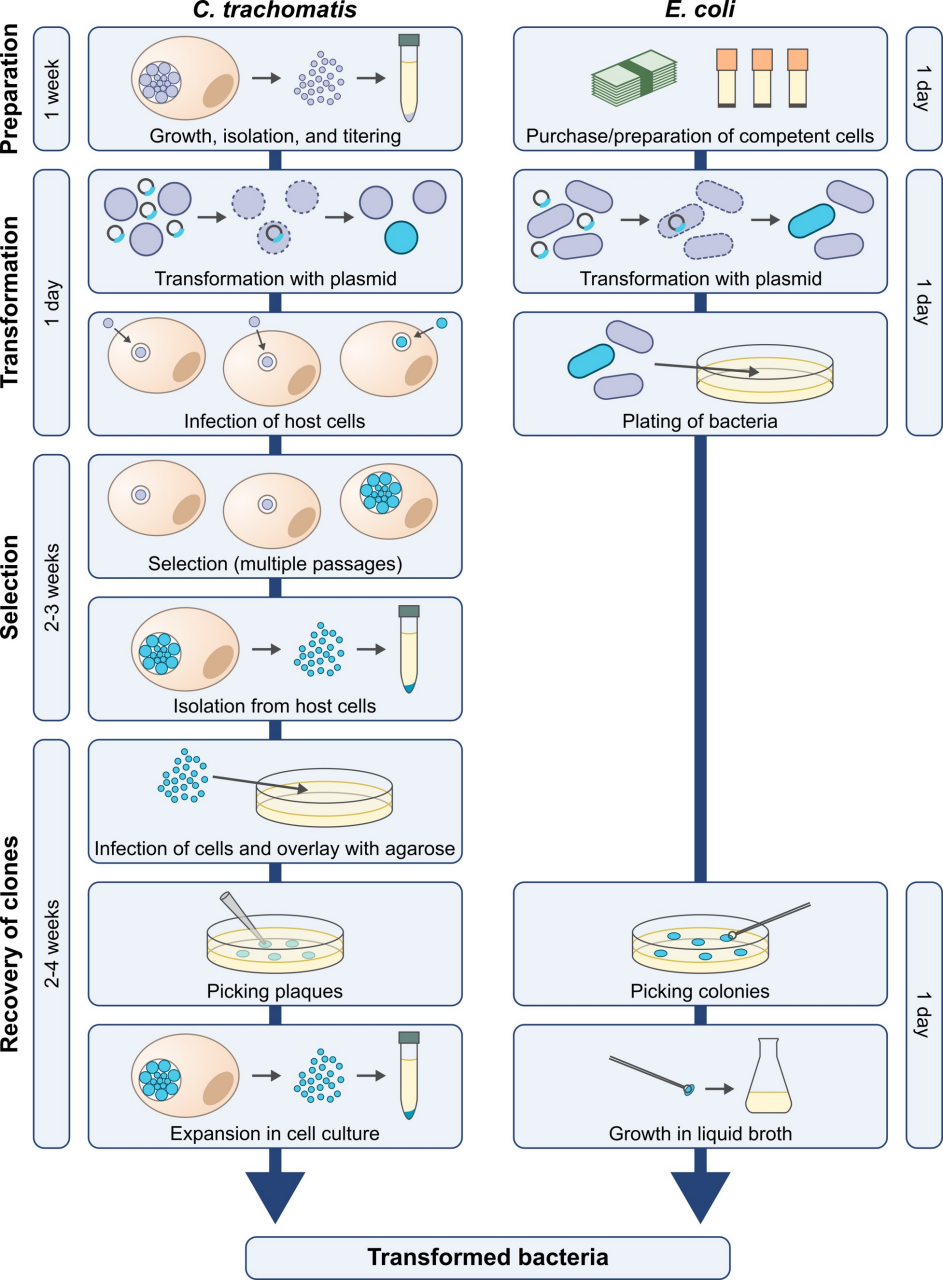
A timeline for the generation of transformed bacteria in *C*. *trachomatis* and *E*. *coli*.

Overall, while becoming technically increasingly feasible, the genetic manipulation of obligate intracellular bacteria remains laborious and time-consuming. For example, clones of transformed *Escherichia coli* may be obtained overnight, while the generation of plaque-purified transformed *Chlamydia* spp. takes at least a month (Fig 1). Moreover, the genetic manipulation of obligate intracellular bacteria is not only complicated by technical difficulties, but also by the fact that many genes with roles in host–pathogen interactions are essential for the ability of these pathogens to replicate and can therefore not be disrupted easily.

Clearly, these hurdles could be overcome if host cell-free cultivation was possible. Indeed, this is well illustrated by the case of *C*. *burnetii*, whose genetic toolbox has been greatly expanded following the development of an axenic medium [19]. Unfortunately, efforts to repeat this feat for other obligate intracellular bacteria have so far only resulted in axenic media that enable survival with detectable metabolic activity [20–22], while media that allow these bacteria to replicate under host cell-free conditions still await development.

## What tools for genetic manipulation of obligate intracellular bacteria have been developed so far?

### Transformation protocols

Genetic manipulation of bacteria often starts with a transformation step in which the bacteria are stimulated to take up and incorporate exogenous DNA. A CaCl_2_-based chemical transformation method has been widely adopted for transformation of host cell-free (purified) *Chlamydia* spp. [4,23–25], while electroporation is the method of choice for other obligate intracellular bacteria [5–7]. In attempts to bypass the need for purified bacteria, polyamidoamine dendrimers were successfully tested in *Chlamydia* spp. [26,27] and *Anaplasma phagocytophilum* [28] to introduce plasmids into bacteria residing in host cells. Yet, dendrimer-based protocols have not yet been further developed and thus remain at a proof-of-principle stage. Of note, in *Chlamydia trachomatis*, the fact that DNA, including plasmids, can be naturally transferred between coinfecting strains through lateral gene transfer has also been exploited to introduce DNA [29].

### Shuttle vectors for gene expression

Shuttle vectors are plasmids that can be engineered in a convenient system such as *E*. *coli* and then transferred through transformation to the host of interest, where the plasmids are stably maintained to enable expression of genes. Shuttle vectors have been developed for several species of obligate intracellular bacteria with *C*. *trachomatis* currently having one of the most versatile arsenals of vectors. These vectors are commonly based on the endogenous plasmid of *C*. *trachomatis* serovar L2 and contain various selection markers and promoter systems, including such that enable inducible gene expression [4,30,31]. More recently, shuttle vectors for other *Chlamydia* spp. have been developed as well [23–25], and attempts are underway to develop a broad-spectrum vector system [32]. For *Rickettsia* spp., vectors were constructed based on endogenous plasmids found in the spotted fever group species *Rickettsia amblyommatis* and were shown to be stably maintained in members from both the spotted fever and typhus groups of the genus [33,34]. Finally, vectors have also been created for *C*. *burnetii* based on plasmid backbones previously used in *Legionella pneumophila* [35,36] and, more recently, based on an endogenous plasmid from *C*. *burnetii* itself [37].

### Targeted modifications in bacterial chromosomes

Site-specific mutations in genomes of obligate intracellular bacteria have principally been generated by using site-specific transposons, the proprietary TargeTron system, or allelic exchange approaches. A transposon system enabling single copy integration of genes at a specific chromosomal site has for instance been described for *C*. *burnetii* [38], while similar approaches have so far not been established in *Chlamydia* spp. In contrast, the TargeTron system, which takes advantage of the ability of mobile group II introns to insert themselves into the bacterial genome and which in contrast to the transposon system can be engineered to target a specific site of choice, has been widely applied in *C*. *trachomatis*, in particular to disrupt genes coding for secreted effector proteins [39–41]. Beyond *Chlamydia* spp., however, the use of TargeTron in obligate intracellular bacteria has so far been limited to *Ehrlichia chaffeensis* [7] and *Rickettsia rickettsii* [42]. Targeted modification by allelic exchange, a process that replaces a selected stretch of DNA via homologous recombination and enables the most versatile modifications, was enabled in *C*. *trachomatis* by the development of fluorescence-reported allelic exchange mutagenesis (FRAEM) [43]. Its latest implementation, exploiting the Cre-lox system for site-specific recombination, even allows for the markerless deletion of operon-localized genes avoiding polar effects [29]. Allelic exchange has also been successfully used to disrupt genes in *C*. *burnetii* [44], *E*. *chaffeensis* [7], and *Rickettsia prowazekii* [45]. Finally, worth mentioning is that the feasibility of generating conditional knockdowns in *C*. *trachomatis* using CRISPR interference has recently been demonstrated [46,47], which now facilitates the study of essential genes that cannot be disrupted by the approaches mentioned above [48,49].

### Random mutagenesis methods

Bacterial genomes can be randomly mutagenized by various means, but the main methods that have been used for obligate intracellular bacteria so far are transposon mutagenesis and exposure to chemical mutagens. Chemical methods have so far been the mainstay of random mutagenesis in *Chlamydia* spp. [50–53], for which successful Himar1-based transposon mutagenesis was only recently reported [54,55]. The latter has, on the other hand, been more commonly used in other obligate intracellular bacteria, including *C*. *burnetii* [56], *E*. *chaffeensis* [7], *A*. *phagocytophilum* [6], and *R*. *prowazekii* [57]. Both mutagenesis methods allow the generation of mutant libraries, which can be used in forward and reverse genetic approaches, i.e., for the identification of mutants that have desired traits or defects in specific genes. An advantage of the chemical mutagenesis approach is that it can also generate hypomorphic alleles, which can facilitate the study of essential genes. In addition, it often introduces multiple mutations per strain; therefore, fewer mutants may need to be screened. However, associating phenotypes with specific chemically induced point mutations is more tedious compared to the identification of single transposon integration sites. In *Chlamydia*, this typically involves whole-genome sequencing in combination with genetic mapping approaches based on the generation of recombinant strains [51,58]. Moreover, strains with point mutations in specific genes can be found either through whole-genome sequencing of entire collections of mutated strains or through a targeted search approach named TILLING (targeting induced local lesions in genomes) [50,52].

## What has the application of genetic tools taught us so far about *Chlamydia* spp.?

**Fig 2:**
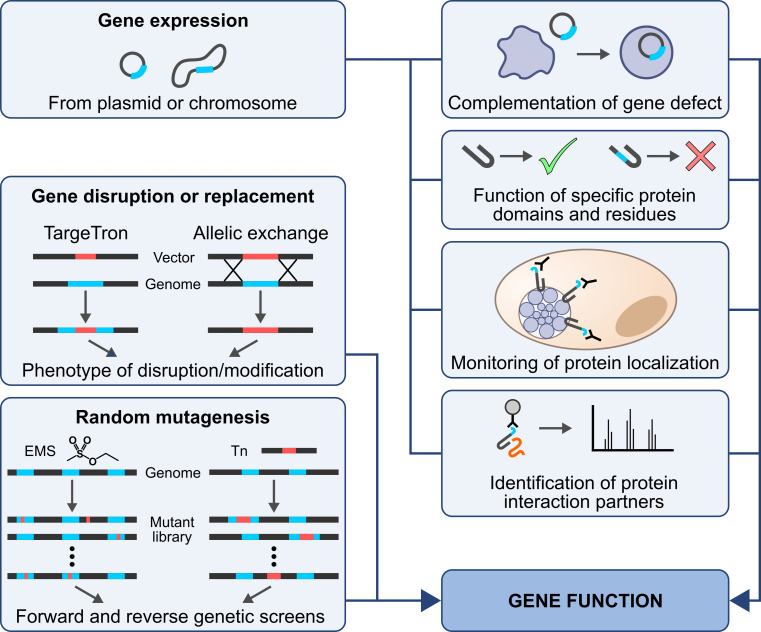
The benefits of a versatile genetic toolbox in the exploration of gene functions.

The ability to disrupt genes and to then complement the observed phenotypic defects by restoring their expression, allows us to unequivocally assign functions to specific genes. Moreover, the expression of tagged proteins facilitates studies of their localization and interactors, while the expression of modified protein variants can help identifying functional domains. Hence, a versatile genetic toolbox is a powerful asset for in-depth gene characterizations (Fig 2).

In *C*. *trachomatis*, the application of these novel approaches has in particular advanced our knowledge of its repertoire of secreted virulence factors and thus our understanding of key aspects of *Chlamydia*’s interaction with host cells. To give examples, two *C*. *trachomatis* effectors, i.e., TarP and TmeA, were shown to promote host cell invasion via remodeling of the actin cytoskeleton at the invasion site [29,59–61]. Moreover, functions could be assigned to a significant number of the so-called inclusion membrane proteins (Incs), a class of secreted effectors that decorate the membrane of the *Chlamydia* vacuole (called inclusion). For instance, IncA was shown to mediate homotypic inclusion fusion [39], IncD and IncV to establish contact sites between the inclusion and the endoplasmic reticulum [62,63], and IncE and CpoS to modulate host membrane trafficking [41,64–66]. CpoS deficiency had a particularly detrimental effect on bacterial replication, as it resulted in a premature death of the infected cell, presumably as a result of premature inclusion lysis [40,41]. Interestingly, inclusion instability was also noted in cells infected with various other Inc mutants [40], including such deficient for InaC, an Inc that promotes microtubule stabilization and actin cage formation at the periphery of the inclusion [52,67,68]. Finally, concerning *Chlamydia* exit from host cells, we learned that the Incs MrcA and CT228 regulate myosin II motor protein activity to promote or suppress exit by extrusion [69,70], while the secreted protease CPAF and the plasmid-encoded factor PGP4 contribute to bacterial egress by host cell lysis [71].

The possibility for genetic manipulation has also empowered the use of reporter genes. For instance, the expression of fluorescent proteins in *Chlamydia* spp., now routinely applied, can ease the analysis of infection-related processes, such as adhesion, entry, and bacterial growth [72]. Moreover, their use for monitoring promoter activities and developmental transitions now also allows us to decode the mysteries of *Chlamydia*’s developmental biology, which, for example, recently revealed that the transition of the replicative form of *C*. *trachomatis* into the infectious form is regulated by bacteria-intrinsic not external cues [73]. In addition, it was shown that the use of luciferase-expressing *Chlamydia muridarum* strains can enable in vivo imaging of *Chlamydia* infections in mice [74]. The application of this novel tool uncovered an unanticipated spread of the bacteria from the primary infection site, the genital tract, to other organs, followed by a long-lasting colonization of the mouse intestine [75]. Of note, the plasmid-encoded effector PGP3 and several chromosomal-encoded bacterial genes were later identified as virulence factors necessary for *C*. *muridarum* to survive the acidic conditions in the stomach and to colonize the colon [76–78]. Because these virulence factors were also required for hydrosalpinx induction, intestinal colonization was proposed to be a key event in promoting upper genital tract pathology in infected mice [76,78].

## What further developments can we expect in the future for the genetic manipulation of *Chlamydia* spp.?

In spite of all the progress made in the genetic manipulation of obligate intracellular bacteria, major technical hurdles remain that significantly restrict the biological insights we can gain. For instance, considering the case of *C*. *trachomatis*, it should now principally be feasible to integrate or replace genes or regulatory elements at any desired site in the chlamydial chromosome or to markerlessly remove genes or larger genomic regions. However, in practice, this remains technically difficult, as it requires recovery of rare double cross-over allelic exchange events. This could be significantly eased if counterselectable markers, already described for *C*. *burnetii* [44], would be available for *Chlamydia* spp. as well. The use of temperature-sensitive alleles has recently been proposed as one possible solution [58].

Another expected major milestone in *Chlamydia* will be the development of saturation mutagenesis enabling transposon-insertion sequencing. It appears that many secreted *Chlamydia* effectors are nonessential in cell culture, suggesting either an inbuilt redundancy of effector functions or more likely context-specific roles. For instance, certain functions might be relevant only in specific host species, tissues, or cell types, or only in the context of an in vivo infection, for instance, to promote immune evasion or dissemination. Transposon-insertion sequencing holds great promise in identifying essential genes and genes providing such context-specific fitness benefits. While the low transformation efficiencies in *Chlamydia* have been considered a major obstacle for establishing such approach, the recent development of inducible transposon mutagenesis in *C*. *trachomatis* suggests that it can be bypassed [79].

Finally, a better understanding of *Chlamydia*’s unique developmental biology will also require a further refinement of our capabilities to study the function of essential genes, such as by developing more tightly controlled inducible expression systems to improve conditional gene expression and disruption approaches. Again, most recent developments in the field, such as the description of riboswitches for translational control of gene expression in *Chlamydia* [79,80], provide a highly encouraging perspective.

Taken together, while there are still many obstacles in our way, we can be confident that the establishment and refinement of novel genetic tools for obligate intracellular bacteria, such as *Chlamydia* spp. and beyond, will continue at fast pace. Clearly, these advances will further revolutionize both the ease and depth with which we can decipher the secret ways by which these pathogens modulate host cell biology and cause diseases.
